# Familial Tooth Agenesis in Lebanese Patients: Clinical Characterization and Whole-Exome Identification of Rare CACNA2D2 and TRIO Variants

**DOI:** 10.7759/cureus.108369

**Published:** 2026-05-06

**Authors:** Fidele Nabbout, Joelle EL Hajj, Bassam Badran, Michella Ghassibe

**Affiliations:** 1 Department of Orthodontics, Faculty of Dental Medicine, Lebanese University, Beirut, LBN; 2 Department of Natural Sciences, School of Arts and Sciences, Lebanese American University, Beirut, LBN; 3 Laboratory of Cancer Biology and Molecular Immunology, Faculty of Sciences I, Lebanese University, Beirut, LBN

**Keywords:** cacna2d2, calcium channels, familial genetics, hypodontia, lebanese population, oligodontia, rhogef rare cacna2d2 and trio variants, tooth agenesis, trio, whole exome sequencing

## Abstract

Introduction: Tooth agenesis (TA) is a common developmental anomaly of the dentition with marked phenotypic and genetic heterogeneity, yet data from Middle Eastern families are limited. The aims of this study were twofold: first, to describe the clinical features and inheritance patterns of familial TA in Lebanese kindreds and to investigate rare segregating exonic variants that may contribute to these phenotypes; and second, to provide a clinic‑based estimate of TA prevalence and patterns in the same population context.

Methods: Records of 157 consecutive patients attending an orthodontic and pediatric dental clinic in Akkar, Lebanon (2015-2019), were reviewed for TA, excluding third molars, to obtain a descriptive, clinic‑based prevalence estimate. In parallel, from a larger craniomaxillofacial cohort, two multigenerational families with TA across at least two generations were recruited. Detailed clinical and radiographic assessments were performed. Whole‑exome sequencing (WES) was undertaken in five affected individuals. Variants were filtered by population frequency, predicted functional impact, and segregation, and conservation was assessed by multispecies protein sequence alignment.

Results: TA was identified in 13.1% of clinic patients (95% confidence interval (CI) 8.4-17.8%). In family 1, six individuals had familial TA without a confirmed syndromic diagnosis, and WES revealed a heterozygous triple functional domain protein (TRIO) missense variant c.8312C>T (p.Ser2771Leu) in exon 53 that co‑segregated with TA in sequenced members. In family 2, five relatives with TA carried a heterozygous calcium voltage‑gated channel auxiliary subunit alpha2delta 2 (CACNA2D2) missense variant c.284G>A (p.Arg95His) in exon 2, which, likewise, co‑segregated with the phenotype in sequenced members. Ser2771 in TRIO and Arg95 in CACNA2D2 lie in highly conserved regions within the immunoglobulin‑like I‑set domain and adjacent to the von Willebrand factor A (VWA) domain, respectively. The TRIO change was absent from the Genome Aggregation Database (gnomAD), whereas the CACNA2D2 variant corresponded to rs149979955, a very rare allele previously annotated as likely benign.

Conclusions: This clinic‑based cohort provides a local descriptive epidemiologic context for TA, and the two Lebanese families broaden the clinical and putative genetic spectrum of familial TA. The rare segregating variants identified in TRIO and CACNA2D2 support these genes as candidate contributors to familial TA without a confirmed syndromic diagnosis; however, additional functional and genetic studies are needed before they can be considered for routine diagnostic use.

## Introduction

Tooth agenesis (TA), the congenital absence of one or more permanent teeth, is among the most common developmental anomalies of the craniofacial complex, with prevalence (excluding third molars) typically ranging from about 3% to 10% depending on ethnicity and sampling strategy. Isolated TA is usually more frequent than syndromic forms and can be sporadic or familial, with autosomal dominant, autosomal recessive, or X‑linked inheritance patterns described [1-3]. The mandibular second premolars, maxillary lateral incisors, and maxillary second premolars are most frequently affected [4-7]. Regional clinic-based studies have also documented variable frequencies of TA and associated dental anomalies in Middle Eastern populations [8,9]. Tooth development relies on tightly regulated epithelial‑mesenchymal interactions progressing through initiation, bud, cap, and bell stages, under the control of signaling pathways such as Wnt signaling (WNT), bone morphogenetic protein (BMP), fibroblast growth factor (FGF), and Sonic hedgehog (SHH) and multiple transcription factors, so disturbances at any stage, including those affecting mineralization, may manifest as TA. Pathogenic variants have been identified in several genes (for example, msh homeobox 1 (MSX1), paired box 9 (PAX9), axis inhibition protein 2 (AXIN2), Wnt family member 10A (WNT10A), and ectodysplasin A (EDA)), with WNT10A accounting for a substantial proportion of selective TA, yet many families remain genetically unexplained. Data from Middle Eastern populations are limited [10-16]. Given that calcium‑dependent processes and ion channels are central to dentin and enamel formation and that triple functional domain protein (TRIO) and calcium voltage‑gated channel auxiliary subunit alpha2delta 2 (CACNA2D2) encode, respectively, a multidomain Rho guanine nucleotide exchange factor (RhoGEF) involved in cytoskeletal dynamics and craniofacial morphogenesis and an α2δ subunit of voltage‑gated calcium channels, both genes represent biologically plausible candidates for TA [17-21].

Objectives

The aim of this study was to characterize the clinical phenotype and underlying genetic causes of TA in two Lebanese multigenerational families and to investigate whether rare variants in TRIO and CACNA2D2 contribute to familial TA. A secondary objective was to estimate the clinic‑based prevalence and pattern of TA in an orthodontic and pediatric dental cohort from the same population, thereby providing region‑specific epidemiological data to complement the familial genetic findings [4-8]

## Materials and methods

Study design and participants

This observational genetic study was embedded within a craniomaxillofacial research program at the Lebanese American University, Beirut, Lebanon, and included a clinic‑based prevalence component and a family‑based exome analysis. For the prevalence analysis, clinical charts and panoramic radiographs of 157 consecutive patients attending a private orthodontic and pediatric dental clinic in Akkar, Lebanon, between 2015 and 2019 were retrospectively reviewed. The 157 patients (68 males, 89 females) had a mean age of 14.2 ± 3.8 years (range: eight to 25 years) and were included only if panoramic radiographs and/or periapical films were available to assess tooth presence or absence. All permanent teeth, except third molars, were evaluated for congenital absence. From a larger research cohort of more than 200 individuals with orofacial dysmorphic features, two unrelated Lebanese families were identified in which TA segregated in at least two generations, and eight family members (six affected and two unaffected) were enrolled for detailed phenotyping and genetic investigation, consistent with family-based approaches used in studies of oligodontia and hypodontia genetics [12,13].​

The study protocol was approved by the Institutional Review Board (IRB) and the Committee on Human Subjects in Research of the Lebanese American University (approval number: LAU.SAS.MS1; approval date 23 May 2018) and adhered to the Declaration of Helsinki. Written informed consent was obtained from all adult participants and from parents or legal guardians of minors, with assent from children when appropriate.

Clinical and radiographic assessment

Two trained dentists performed standardized clinical examinations in a dental chair under artificial light. For each participant, the presence or absence of all permanent teeth, excluding third molars, was recorded, and tooth morphology and spacing were assessed; features such as microdontia, peg‑shaped lateral incisors, conical teeth, and generalized spacing were noted. Panoramic radiographs were obtained or retrieved from records for all affected family members and clinic patients as part of routine assessment. TA was diagnosed when a tooth bud was absent on radiographs, and there was no history of extraction, trauma, or impaction, in line with commonly used radiographic criteria in orthodontic TA studies [4,5]. Medical records were reviewed to identify systemic or craniofacial anomalies suggestive of syndromes [14,22].

DNA extraction and whole‑exome sequencing

Peripheral blood samples were collected in ethylenediaminetetraacetic acid (EDTA) tubes from consenting family members, and genomic DNA was extracted from leukocytes using standard phenol‑chloroform protocols. Whole‑exome sequencing (WES) was performed on 1 µg of DNA from five affected individuals (three from family 1 and two from family 2). Exome capture used the SureSelect Human All Exon V6 kit (Agilent Technologies, Inc., Santa Clara, CA, USA), and sequencing was carried out on an Illumina HiSeq 3000 (Illumina, Inc., San Diego, CA, USA), generating 150‑bp paired‑end reads with a mean on‑target depth of approximately 60×. Reads were aligned to the GRCh37 reference genome using the Burrows-Wheeler Aligner (Heng Li, Broad Institute, Cambridge, MA, USA), and variants were called using SAMtools (Heng Li and collaborators; associated with the Wellcome Sanger Institute, Hinxton, Cambridge, UK), Picard (Broad Institute), and the Genome Analysis Toolkit (Broad Institute), with annotation by Variant Effect Predictor (European Bioinformatics Institute, Hinxton, Cambridge, UK), following exome-based strategies previously applied in oligodontia and hypodontia studies [12,13]. Variants were filtered to retain rare coding and splice‑site changes (minor allele frequency <0.001 in Genome Aggregation Database (gnomAD) and other population databases), and prioritization focused on nonsynonymous, splice‑altering, or predicted loss‑of‑function variants that segregated with the TA phenotype within each family. Predicted functional impact was assessed using six in silico tools (SIFT, PolyPhen‑2, MutationTaster, CADD, REVEL, and M‑CAP), and conservation was evaluated by multi‑species protein sequence alignment; for the TRIO c.8312C>T (p.Ser2771Leu) and CACNA2D2 c.284G>A (p.Arg95His) variants, the corresponding allele frequencies, prediction results, and conservation findings are summarized in the Results to clarify the rationale for their retention as candidate contributors. 

Variant filtering and in silico analysis

Variants were filtered to retain rare, potentially damaging changes compatible with autosomal dominant inheritance. Common polymorphisms were excluded based on allele frequencies in the gnomAD, 1000 Genomes, the National Heart, Lung, and Blood Institute (NHLBI) Exome Sequencing Project (ESP6500), and other population datasets using a minor allele frequency cutoff of 1%, as commonly applied in exome studies of familial TA [12,13]. High‑ or moderate‑impact variants according to SnpEff were retained, and genes with high intolerance to loss‑of‑function or missense variation were prioritized using Exome Aggregation Consortium (ExAC) constraint metrics. Missense variants were considered strong candidates when at least three of six prediction tools suggested a deleterious effect.

Copy number variation (CNV) was assessed from exome read depth using ExomeDepth (University of Cambridge, Cambridge, UK) to detect exonic deletions or duplications potentially co‑segregating with TA. For the TRIO and CACNA2D2, conservation of the affected amino acids and surrounding domains was evaluated by aligning protein segments across multiple vertebrate species using the Basic Local Alignment Search Tool (National Center for Biotechnology Information (NCBI), Bethesda, MD, USA), HomoloGene (NCBI), and Constraint-Based Multiple Alignment Tool (NCBI). Gene structure and domain organization were retrieved from NCBI Gene (NCBI), UniProt (European Bioinformatics Institute, Hinxton, UK; Swiss Institute of Bioinformatics, Lausanne, Switzerland; and Protein Information Resource, Washington, DC, USA), and Protein Families Database (European Bioinformatics Institute), with functional interpretation guided by prior work on TRIO and CACNA2D2 domains [18,21]. Variant interpretation followed American College of Medical Genetics and Genomics/Association for Molecular Pathology (ACMG/AMP)‑style principles, with emphasis on rarity, predicted deleteriousness, segregation within families, and location in functionally important protein domains [11,23].

Statistical analysis

For the clinic cohort, the prevalence of TA excluding third molars was calculated as the proportion of patients with at least one congenitally missing permanent tooth, and a 95% confidence interval (CI) was computed using the exact binomial method. Descriptive statistics summarized the number and distribution of missing teeth by jaw and tooth type. In the family study, segregation of candidate variants was assessed qualitatively, given the limited pedigree sizes; formal linkage analysis was not performed. All analyses used standard statistical software. Given the exploratory and descriptive nature of the study and the limited sample size, no formal hypothesis‑testing or multivariable modeling was undertaken, and results are presented as descriptive statistics with corresponding CIs where appropriate.

## Results

Prevalence of TA in the clinic cohort

Among 157 orthodontic and pediatric patients, 21 (13.1%) had at least one congenitally missing permanent tooth other than third molars (Table 1). The 95% CI for this proportion, based on the exact binomial method, was 8.4%-17.8%. This estimate lies within the upper range of prevalence reported in similar orthodontic samples worldwide [4-8,23]. Most affected individuals had hypodontia involving one to four teeth, typically mandibular second premolars and maxillary lateral incisors, consistent with prior epidemiologic reports [8-10]. No clear syndromic pattern was observed in the clinic cohort, and most cases appeared isolated, similar to the predominance of non-syndromic presentations described in the literature [14,22,24]. No formal hypothesis‑testing was performed, and findings are reported as descriptive estimates with 95% CIs where applicable.

**Table 1:** Prevalence and pattern of tooth agenesis (TA) in the Lebanese orthodontic and pediatric clinic cohort. Summary of the number of patients examined, proportion with TA excluding third molars, confidence interval (CI) for prevalence, mean number of missing teeth among affected individuals, most commonly missing tooth types, and whether cases appeared syndromic or non‑syndromic. FDI World Dental Federation (FDI) tooth numbering is used for tooth codes. *Values are calculated only among patients with TA in the clinic cohort.

Variable	Value
Total patients reviewed	157
Patients with TA* (≥1 missing tooth, excluding third molars)	21 (13.1%)
95% CI for TA prevalence	8.4–17.8%
Mean number of missing teeth in TA cases*	2.8 (range 1–8)*
Predominant pattern*	Hypodontia (1–4 teeth)
Most frequently missing teeth*	Mandibular second premolars (FDI 35, 45)
Most frequently missing teeth*	Maxillary lateral incisors (FDI 12, 22)
Most frequently missing teeth*	Maxillary second premolars (FDI 15, 25)
Syndromic cases	None clearly identified; TA mostly isolated

Family 1: clinical findings and the TRIO variant

**Figure 1:**
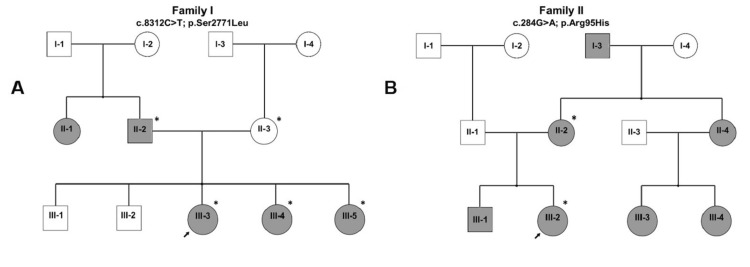
Pedigrees of the two Lebanese families with familial tooth agenesis (TA). (A) Pedigree of family 1 showing six individuals with hypodontia/oligodontia across three generations; affected individuals are indicated by filled symbols, and carriers of the heterozygous triple functional domain protein (TRIO) c.8312C>T (p.Ser2771Leu) variant are marked. (B) Pedigree of family 2 showing six individuals with TA in three generations; affected individuals are indicated by filled symbols, and carriers of the heterozygous calcium voltage‑gated channel auxiliary subunit alpha2delta 2 (CACNA2D2) c.284G>A (p.Arg95His) variant are marked. Squares represent males, circles represent females, and diagonal slashes indicate deceased individuals. An asterisk indicates individuals who underwent whole‑exome sequencing (WES). The schematic was generated using Microsoft PowerPoint (Microsoft Corp., Redmond, WA, USA).

Family 1 was a non‑consanguineous Lebanese pedigree with six affected members across three generations (Table 2, Figure 1A). The proband (III‑3) showed oligodontia with eight missing permanent teeth and no systemic findings. Two sisters (III‑4, III‑5) had hypodontia with four and six missing teeth, respectively; their father and paternal aunt also lacked multiple teeth. One affected sister had additional facial anomalies involving ears, eyes, cheekbones, and chin, but no formal syndrome was diagnosed. A panoramic radiograph of the proband illustrates the pattern of oligodontia.​

**Table 2:** Clinical and genetic characteristics of affected and unaffected members in the two Lebanese families with familial tooth agenesis (TA). Overview of family membership, sex, TA status, number, and distribution of missing permanent teeth, and genotypes for the TRIO c.8312C>T (p.Ser2771Leu) and CACNA2D2 c.284G>A (p.Arg95His) variants. F: female; M: male; TRIO: triple functional domain protein; CACNA2D2: calcium voltage‑gated channel auxiliary subunit alpha2delta 2; FDI: FDI World Dental Federation

Family	Individual ID	Sex	Affected (TA)	Number of missing permanent teeth (excludingthird molars)	Main missing teeth (FDI codes)	TRIO c.8312C>T (p.Ser2771Leu)	CACNA2D2 c.284G>A (p.Arg95His)
1	II‑x (father)	M	Yes	5	12, 22, 35, 45, 15	Heterozygous	Wild-type
1	II‑y (paternal aunt)	F	Yes	4	12, 22, 35, 45	Heterozygous	Wild-type
1	III‑3 (proband)	F	Yes	8	12, 22, 15, 25, 35, 45, 31, 41	Heterozygous	Wild-type
1	III‑4	F	Yes	4	12, 22, 35, 45	Heterozygous	Wild-type
1	III‑5	F	Yes	6	12, 22, 15, 25, 35, 45	Heterozygous	Wild-type
1	III‑x (unaffected sib)	F/M	No	0	–	Wild-type	Wild-type
2	II‑1 (mother)	F	Yes	3	14, 34, 46	Wild-type	Heterozygous
2	III‑1	M	Yes	2	12; peg-shaped 22	Wild-type	Heterozygous
2	III‑2 (proband)	M	Yes	2	13, 23	Wild-type	Heterozygous
2	III‑x (unaffected sib)	F/M	No	0	–	Wild-type	Wild-type

WES in three affected relatives identified a rare heterozygous missense variant in TRIO (NM_007118.4: c.8312C>T; p.Ser2771Leu) in exon 53. This change was absent from gnomAD and ClinVar and was not present in major disease databases at the time of analysis [18-20]. Ser2771 lies within the immunoglobulin I‑set domain of TRIO, immediately upstream of a protein kinase‑like region, and multiple sequence alignment showed that Ser2771 and its surrounding domain are highly conserved across vertebrates, consistent with the developmental importance of TRIO in craniofacial biology. Segregation analysis indicated that all genotyped affected members carried the TRIO c.8312C>T variant, whereas available unaffected relatives were non‑carriers, consistent with autosomal dominant inheritance. Taken together, these data support TRIO c.8312C>T (p.Ser2771Leu) as a rare segregating candidate contributor to familial TA in this pedigree. No exonic deletions or duplications in known TA genes were detected by ExomeDepth.

Family 2: clinical findings and the CACNA2D2 variant

**Figure 2:**
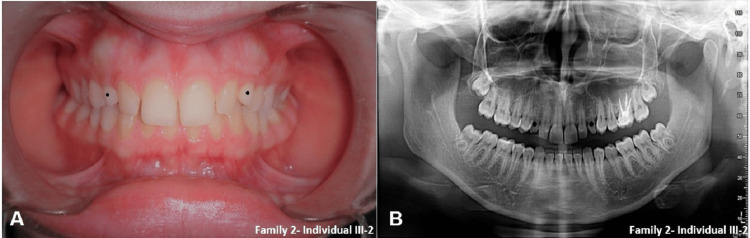
Clinical and radiographic presentation of tooth agenesis in the proband from Family 2. (A) Frontal intraoral photograph showing anterior spacing and absence of the maxillary canines. (B) Panoramic radiograph of the same individual demonstrating bilateral agenesis of the maxillary canines (teeth 13 and 23)

Family 2 is another non‑consanguineous Lebanese kindred in which TA affected six members in three generations (Table 2, Figure 1B). The proband (III‑2) had bilateral agenesis of the maxillary canines (13, 23), confirmed clinically and radiographically (Figure 2). His brother (III‑1) showed agenesis of tooth 12 and a peg‑shaped maxillary lateral incisor [22]. Their mother (II‑1) lacked teeth 14, 34, and 46. None of the examined individuals displayed systemic features or neurological deficits.

WES in two affected individuals revealed a rare heterozygous missense variant in CACNA2D2 (NM_006030.3: c.284G>A; p.Arg95His) in exon 2. This variant corresponds to rs149979955, observed at very low allele frequencies in reference populations and previously annotated as likely benign in ClinVar [21]. Arg95 is located immediately N‑terminal to the von Willebrand factor A (VWA) domain, which contains metal‑binding sites important for CACNA2D2 function. Alignment of residues 83-127 showed strong conservation of Arg95 and its motif across vertebrate species, in keeping with known functional constraints on CACNA2D2‑related calcium‑channel biology 18,21]. Within family 2, the CACNA2D2 variant co‑segregated with TA in all genotyped affected members and was absent in available unaffected relatives, and no CNVs consistent with TA were identified. Given its prior likely benign annotation, these findings support CACNA2D2 c.284G>A (p.Arg95His) as a more tentative, rare segregating candidate contributor that warrants further investigation in familial TA.

## Discussion

This study integrates clinic‑based epidemiological data with family‑based WES to explore the genetic basis of non-syndromic TA in a Lebanese population. We observed a relatively high prevalence of TA in an orthodontic and pediatric dental cohort and identified rare missense variants in TRIO and CACNA2D2 that co‑segregate with TA in two independent multigenerational families, thereby expanding the spectrum of candidate genes that may contribute to familial TA. However, these findings should be regarded as hypothesis‑generating rather than definitive evidence of causality.

The prevalence of TA in our clinic cohort (13.1%; 95% CI 8.4%-17.8%) lies at the upper end of the range typically reported for orthodontic samples, in which TA frequently affects 3% to 10% of individuals depending on ethnicity and ascertainment strategy [4-8,23]. This likely reflects clinic‑based selection, as orthodontic and pediatric dental practices tend to include a higher proportion of patients with dental anomalies compared with the general population [3-7]. Accordingly, our prevalence estimate should not be generalized to the broader Lebanese population but is consistent with reports from similar orthodontic cohorts in other regions [4-8,23]

In both families, the observed inheritance pattern is compatible with autosomal dominant transmission with variable expressivity, which is well recognized in nonsyndromic TA [14,22,24]. The variability in the number and distribution of missing teeth among affected relatives, including presentations ranging from mild hypodontia to oligodontia and the presence of additional craniofacial features in a single individual from family 1, supports a multifactorial model in which modifier genes and environmental factors modulate expressivity and penetrance [12-14,23-25]. This is in line with recent reviews emphasizing oligogenic and polygenic contributions to TA beyond single‑gene Mendelian models [24,25].

Importantly, WES did not reveal pathogenic or likely pathogenic variants in established TA‑associated genes such as MSX1, PAX9, AXIN2, WNT10A, and EDA [23-25]. This negative finding is consistent with the observation that a substantial proportion of familial TA remains genetically unexplained even after targeted testing of major loci, particularly in under‑studied populations [23-25]. Within this context, the identification of rare missense variants in TRIO and CACNA2D2 in two unrelated Lebanese pedigrees provides biologically plausible, though still preliminary, candidate contributors to the phenotype.

**Figure 3:**
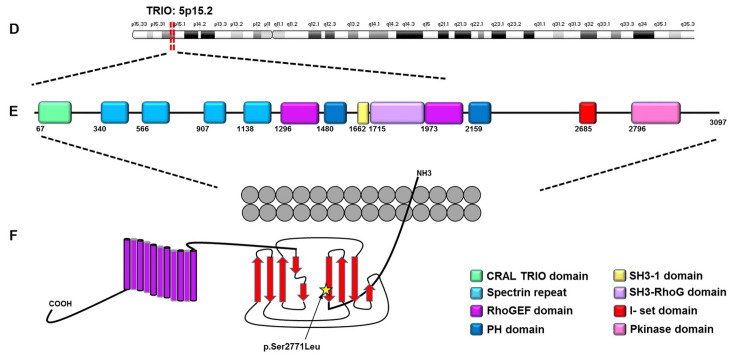
Schematic representation of the triple functional domain protein (TRIO) protein showing its main functional domains and the position of the heterozygous p.Ser2771Leu variant within the intracellular region. Schematic representation of the TRIO protein showing its main functional domains and the position of the heterozygous p.Ser2771Leu variant within the intracellular region. The CRAL_TRIO domain is shown in green, spectrin repeats in blue, Rho guanine nucleotide exchange factor (RhoGEF) domains in magenta, pleckstrin homology (PH) domains in dark blue, Src homology 3‑1 (SH3‑1) domain in yellow, SH3‑RhoG domain in light purple, immunoglobulin I‑set domain in red, and kinase domain in pink. The schematic was recreated manually using Microsoft PowerPoint (Microsoft Corp., Redmond, WA, USA).

The TRIO variant (c.8312C>T; p.Ser2771Leu) affects a highly conserved residue within the immunoglobulin I‑set domain of the protein, immediately upstream of a protein kinase‑like region (Figure 3). TRIO encodes a multidomain RhoGEF involved in cytoskeletal organization, cell migration, and craniofacial development, and experimental work has implicated TRIO in tooth‑root development via p38 mitogen‑activated protein kinase (MAPK) signaling [19]. Previous reports have linked TRIO loss‑of‑function and missense variants to neurodevelopmental disorders with craniofacial and dental anomalies, further supporting its relevance to craniofacial biology [20]. The absence of the p.Ser2771Leu variant from the gnomAD and ClinVar, together with strong evolutionary conservation of the affected residue and domain, supports its potential functional importance; however, in the absence of functional data, its causal role in TA remains speculative [26-28].

**Figure 4:**
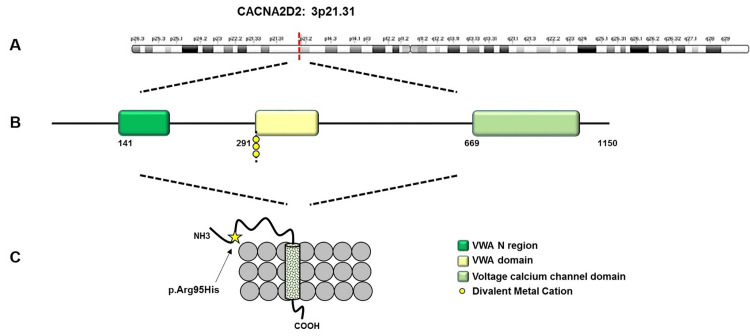
Schematic representation of the calcium voltage‑gated channel auxiliary subunit alpha2delta 2 (CACNA2D2) protein showing its main functional domains and the position of the heterozygous p.Arg95His variant within the N‑terminal region. (A) Chromosomal localization of CACNA2D2 on chromosome 3p21.31. (B) Linear domain organization of the CACNA2D2 α2δ‑2 subunit showing the von Willebrand factor A (VWA) N‑region, VWA domain, and voltage calcium channel domain; yellow circles indicate predicted divalent metal cation‑binding sites. (C) Enlarged schematic of the N‑terminal region illustrating the position of the heterozygous p.Arg95His variant (yellow star) within the extracellular segment of the protein. The schematic was recreated manually using Microsoft PowerPoint (Microsoft Corp., Redmond, WA, USA).

The CACNA2D2 variant (c.284G>A; p.Arg95His) identified in family 2 lies immediately N‑terminal to the VWA domain of the α2δ‑2 subunit of voltage‑gated calcium channels, a region important for channel function and metal binding (Figure 4) [18,21]. Calcium signaling is critical for odontogenesis and mineralization, and CACNA2D2 has been implicated in neurological channelopathies, making it a biologically plausible candidate for dental phenotypes [17,18,21]. Nonetheless, this variant corresponds to rs149979955, a very rare allele previously annotated as likely benign in ClinVar, which warrants particular caution in interpretation despite its rarity, conservation, and co‑segregation with TA in this family [21,27]. It is possible that p.Arg95His acts as a low‑penetrance risk allele or modifier in a polygenic background, but chance co‑segregation cannot be excluded on the basis of the current data. Overall, the TRIO c.8312C>T (p.Ser2771Leu) variant currently appears more compelling as a rare segregating candidate contributor to familial TA, given its co‑segregation, absence from population databases, and location in a conserved functional domain, whereas the CACNA2D2 c.284G>A (p.Arg95His) variant should be regarded as a more tentative candidate in light of its prior likely benign annotation and the available evidence.

In both pedigrees, co‑segregation of the TRIO and CACNA2D2 variants with the TA phenotype was observed among the limited number of genotyped individuals, and unaffected relatives tested so far did not carry the variants. However, the small pedigree sizes, limited number of informative meioses, and lack of formal linkage analysis restrict the strength of segregation evidence. According to ACMG/AMP criteria, such data provide at most supporting evidence for segregation (PP1), and in combination with rarity and in silico predictions (PM2, PP3), both variants are best classified as variants of uncertain significance rather than established pathogenic alleles [11,23-26].

Taken together, our findings raise the possibility that alterations in RhoGEF‑mediated cytoskeletal signaling (via TRIO) and calcium‑channel regulation (via CACNA2D2) may contribute to molecular pathways underlying tooth development in a subset of familial TA [17-19,21]. These pathways are biologically interconnected and relevant to epithelial‑mesenchymal interactions, cell proliferation and differentiation, and enamel and dentin mineralization, all of which are known to be disrupted in dental developmental anomalies [17,18,22]. Nevertheless, the current evidence does not establish a direct mechanistic link between the specific variants identified here and TA, and functional validation in relevant dental cell types or animal models will be required to test these hypotheses [17-19,21,28].

This study has several strengths, including detailed clinical and radiographic characterization of affected individuals, use of a family‑based sequencing design, and inclusion of patients from a Middle Eastern population that remains underrepresented in genetic studies of dental anomalies [23-25]. At the same time, important limitations must be acknowledged. The sample size is small, involving only two families and a modest clinic cohort, which limits statistical power, generalizability, and the ability to estimate penetrance or detect modest genotype-phenotype correlations [14,23-25]. No functional assays were performed to assess the biological impact of the TRIO and CACNA2D2 variants, even though functional studies are increasingly recognized as critical for variant interpretation and reclassification. Moreover, orthogonal confirmation by Sanger sequencing was not feasible for all relatives, and our copy number variant analysis has limited sensitivity for small events, so additional rare variants or structural changes may have been missed. WES‑based approaches also do not capture non‑coding regulatory changes, deep intronic variants, or some structural rearrangements that may contribute to TA, and environmental influences and potential oligogenic interactions with other dental‑development genes were not systematically evaluated [11,23-25].

Future research should aim to validate and extend these findings through several complementary strategies. Functional studies of the TRIO p.Ser2771Leu and CACNA2D2 p.Arg95His variants in dental or craniofacial cell models, as well as in vivo animal systems, are needed to clarify their impact on signaling pathways and tooth development and align with emerging recommendations emphasizing the value of well‑designed functional assays for variant interpretation [26,28]. Replication of similar TRIO and CACNA2D2 variants in independent cohorts with familial TA, ideally with larger pedigrees allowing robust segregation and linkage analyses, would strengthen the case for their involvement [23-25]. Broader genomic approaches, including whole‑genome sequencing and systematic assessment of structural variants and non‑coding regulatory regions, may help capture additional contributors to the marked genetic heterogeneity of TA, particularly in under‑studied populations [23-25].

From a clinical perspective, our results underscore the importance of careful dental and craniofacial phenotyping, as well as systematic family history assessment, in patients with TA, as these steps can guide targeted genetic investigation and counseling [11,22]. Given the current level of evidence, it would be premature to recommend routine inclusion of TRIO and CACNA2D2 in diagnostic gene panels for TA, although these genes may reasonably be considered in research‑oriented sequencing pipelines or exploratory analyses aimed at expanding the molecular spectrum of the condition [22-25]. As additional functional and genetic evidence accumulates, the classification and clinical relevance of TRIO‑ and CACNA2D2‑related variants for TA may be refined, potentially informing more precise risk assessment and personalized management in affected families [22-26,28].

## Conclusions

In a Lebanese orthodontic and pediatric dental clinic, TA affected approximately one in eight patients when third molars were excluded, representing a clinic‑based prevalence estimate rather than a population‑wide figure. In two multigenerational Lebanese families, rare, conserved missense variants in TRIO and CACNA2D2 co‑segregated with familial TA without a confirmed syndromic diagnosis and mapped to functionally important protein regions, supporting these genes as candidate contributors to familial TA but not yet as definitively causal loci. Further functional and genetic studies in larger cohorts will be required to clarify the mechanistic roles, penetrance, and potential clinical utility of TRIO‑ and CACNA2D2‑related variants in TA.
